# Screening Antitumor Compounds Psoralen and Isopsoralen from *Psoralea corylifolia* L. Seeds

**DOI:** 10.1093/ecam/nen087

**Published:** 2011-03-10

**Authors:** Yi Wang, Chengtao Hong, Chenguang Zhou, Dongmei Xu, Hai-bin Qu

**Affiliations:** Department of Chinese Medicine Science & Engineering, College of Pharmaceutical Sciences, Zhejiang University, Hangzhou, China

## Abstract

*Psoralea corylifolia* L. (Fabaceae) is a widely used medical plant in China. This study was designed to screen and identify bioactive compounds with anticancer activity from the seeds of *Psoralea corylifolia* L. One volatile fraction (fraction I) and three other fractions (fraction II, III, IV) from methanol extraction of *P. corylifolia* L. were obtained. Bioactivities of these fractions were evaluated by the cytotoxicity on KB, KBv200, K562, K562/ADM cancer cells with MTT assay. Major components in the active fraction were identified by HPLC/MS^n^. Fraction IV significantly inhibits the growth of cancer cells in a dose-dependent manner. The IC_50_ values were 21.6, 24.4, 10.0 and 26.9, respectively. Psoralen and isopsoralen, isolated from fraction IV, were subject to bioactive assay and presented a dose-dependent anticancer activity in four cancer cell lines (KB, KBv200, K562 and K562/ADM). The IC_50_ values of psoralen were 88.1, 86.6, 24.4 and 62.6, which of isopsoralen were 61.9, 49.4, 49.6 and 72.0, respectively. Apoptosis of tumor cell significantly increased after treated with psoralen and isopsoralen. Induction of apoptotic activity was confirmed by flow cytometry after staining with Annexin V/PI. These results suggested psoralen and isopsoralen contribute to anticancer effect of *P. corylifolia* L.

## 1. Introduction

The seeds of *Psoralea corylifolia* L. (Fabaceae), well-known as traditional Chinese medicine “Buguzhi”, are widely used for the treatment of various kinds of disorders such as asthma, cough, nephritis, vitiligo and calvities [1, 2]. The constituents in *P. corylifolia* L. include coumarins and flavone components, such as psoralen, isopsoralen, psoralidin, neobavaisoflavone, bavachin, corylin, bavachalcone, and so forth [3]. It has been reported that the methanol (MeOH) extract of *P. corylifolia* L. showed remarkable inhibitory effects on NO production [4]. The cytotoxic, anticancer and immunomodulatory properties of *P. corylifolia* L. seeds have already been reported [5]. In addition, the active fraction from both roots and seeds of *P. corylifolia* L. exhibited cytotoxicity against cultured human cancer cell lines [6, 7]. However, to the best of our knowledge, the bioactive compounds from the seed of *P. corylifolia* L., which are potential to inhibit cancer cell proliferation *in vitro*, have not yet been evaluated.

The aim of the present study was to screen and identify active compounds with anticancer effect from the seeds of *P. corylifolia* L. Bioassay-guided isolation was performed to obtain two active compounds from MeOH extracts of *P. corylifolia* L. seeds. Psoralen and isopsoralen exhibited dose-dependent anticancer effect on four (KB, KBv200, K562, K562/ADM) cell lines.

## 2. Methods

### 2.1. Plant Materials and Reagents

The crude material of *P. corylifolia* L. seed was purchased from Hangzhou Traditional Chinese Medicine Company (Hangzhou, China). The crude sample was authenticated morphologically by Prof. He Qing (College of Pharmaceutical Sciences, Zhejiang University). A voucher specimen was deposited in the herbaria (Department of Chinese Medicine Sciences & Engineering, Zhejiang University, China), under the acquisition number of GZ. Standards of psoralen and isopsoralen were purchased from National Institute for the Control of Pharmaceutical and Biological Products (Beijing, China).

MeOH and EtOH were analytical grade from Hangzhou Reagent Company (Hangzhou, China). HPLC-grade acetonitrile and DMSO were from E. Merck (Darmstadt, Germany). Distilled water used in all experiments was purified by a Milli-Q system (Milford, MA, USA). DMEM, RPMI-1640 medium, fetal bovine serum and non-essential amino acids were purchase from Gibco (CA, USA). Trypsin was obtained from Sigma (St Louis, USA). Newborn calf serum was purchased from SeRa Biotech Co. Ltd (Hangzhou, China).

Vincristine sulfate (Hanye Pharmaceutical Co. Ltd, Guangzhou, China) and doxorubicin hydrochloride (Haizheng Hisun Pharmaceutical Co. Ltd, Zhejiang, China) were used as positive control.

### 2.2. Extraction and Isolation

Air-dried plant material (500 g) was powdered and macerated with 2 L distilled water overnight. The solution was Soxhlet's extracted for 5 h to obtain volatile components as fraction I. The filtered deposit was extracted with 2 L 95% EtOH solution for 2 h. The extraction was filtrated and evaporated at 60°C in vacuum. The extract was subjected to an ODS-C_18_ column for further separation with a step gradient of MeOH aqueous solution. The elution of 30, 50, and 70% methanol was collected to obtain fraction II, III, and IV.

Further separations of fraction IV were performed on a RP-18 semi-preparative column (5 *μ*m, 10 mm i.d. × 25 cm; Hanbang Science & Technology Co., Jiangsu, China) and Agilent 1100 series preparative HPLC system (Waldbronn, Germany) equipped with a binary high-pressure mixing pump and fraction collector.

### 2.3. Cell Culture

Human oral carcinoma line KB, KBv200 (the vincristine resistance subline of KB), human erythroleukemia cell K562 and K562/ADM (the doxorubicin resistance subline of K562) were kindly gifted by Prof. Yang Bo (College of Pharmaceutical Science, Zhejiang University), and human periodontal ligament (HPDL) cell were gifted by Prof. Hua Fu (College of Medicine, Zhejiang University). K562 and K562/ADM were cultured in RPMI-1640 medium, whilst KB and KBv200 were in DMEM; supplemented with 10% heat-inactivated newborn calf serum and 1% non-essential amino acids; HPDL cells were cultured in DMEM, supplemented with 10% heat-inactivated fetal bovine serum, all with 100 U mL^−1^ penicillin–streptomycin. All the cells were incubated at 37°C in a humidified atmosphere of 95% air and 5% CO_2_ and passaged three times a week. After passaged four times, HPDL cells could be used as normal human cells.

### 2.4. Antitumor Activity Assays

The cytotoxity was analyzed by the 3-(4,5-dimethylthiazol-2-yl)-2.5-diphenyl tetrazolium bromide (MTT) assay. In this assay, cancer cells were seeded at cell density of 2 × 10^3^ per well in 96-well round-bottom test plate and incubated for 24 h before test. To examine the anticancer effects of the *P. corylifolia* L. fractions, the K562 and KB cells were incubated with different fractions (80 *μ*g mL^−1^). Each fraction was repeated four times. Cells treated with 0.1% DMSO were included as negative control. Vincristine sulfate and doxorubicin hydrochloride, two clinically used anticancer drugs, were used as positive control [8]. After 48-h incubation, 20 *μ*L MTT solution (5 mg mL^−1^) was added to each well and incubated at 37°C for 4 h. After removing the medium, the formazan was dissolved in DMSO and the optical density was measured at 550 nm using an ELx800 UV universal microplate reader (Bio-Tek, USA). The inhibition rate was calculated using [1]:

(1) $$\text{Inhibition}\;\text{ Rate}=\frac{1-(\text{Sample}\;\text{ OD}-\text{Blank}\;\text{ OD})}{\left(\text{Control}\;\;\text{ OD}-\text{Blank}\;\text{ OD}\right)}.$$

Similarly, anticancer effect of fraction IV, psoralen and isopsoralen was assayed on K562, K562/ADM, KB, KBv200, and HPDL cells by the MTT assay. IC_50_ was calculated from dose-inhibition curves.

## 3. Identification of Active Compounds in Fraction IV

Preliminary phytochemical investigations of the fractions were carried out by HPLC/MS^n^. The chromatographic separation was performed on a Agilent Zorbax SB-C_18_ column (5 *μ*m, 4.6 mm i.d. × 25 cm, Hanbang Science & Technology Co., Jiangsu, China) with an Agilent 1100 HPLC system (Waldbroonn, Germany) equipped with a quaternary pump, vacuum degasser, autosampler, diode-array detector, column heater-cooler and ChemStation system. A LCQ DECA XP Plus mass spectrometer (Thermo Finnigan, San Jose, USA) equipped with an ESI interface and an ion trap mass analyzer were used to carry out the MS and MS^n^. The mobile phase consisted of two eluents: water (W) and acetonitrile (A). The flow rate was set at 0.8 mL min^−1^ and the following gradient was used: initial 75% W and 25% A; 25-min linear change to 65% W and 35% A; 25-min linear to 50% W and 50% A; 10-min linear to 30% W and 70% A. The column temperature was set at 30°C. UV detection was set at 300 nm and spectrograms were recorded between 190 and 400 nm at the apex of each peak. By comparison of molecular weight, retention time, UV absorbance and mass fragments information with those of published data and the standards, a number of compounds were tentatively identified.

### 3.1. Morphological Studies of Apoptotic Cell

KB cells from exponentially growing cultures were seeded within 96-well microplate and incubated for 24 h. Then cells were treated with vincristine sulfate, psoralen or isopsoralen and for 48 h. After treatment, cells were washed with PBS once. Cells were investigated using phase contrast microscope (Leika DMI6000, USA). And then cells were stained with Hoechst 33342 dye assay (Beyotime Inst. Biotech.) for 15 min at room temperature before examining under a fluorescent microscope (Leika DMI6000, USA) with an excitation wavelength of 365 nm [9].

### 3.2. Detection of Apoptosis by Annexin V-EGFP/PI Staining

KB cells (5 × 10^5^) were seeded within 6-well plate and incubated for 24 h before treated with vincristine sulfate, psoralen or isopsoralen and for 48 h. After treatment cells were harvested by 0.25% (w/v) Trypsin-0.53 mM EDTA solution. The detection of apoptosis was measured with the Annexin V-FITC Apoptosis Detection Kit I (BD Biosciences). Specific binding of Annexin V-FITC was performed by the incubation of cells for 15 min at room temperature in the dark, in a binding buffer (10 mM Hepes/NaOH (pH 7.4), 140 mM NaCl, 2.5 mM CaCl_2_) containing a saturating concentration of annexin V-fluorescein isothiocyanate (FITC) and propidium iodide (PI) [9, 10]. After incubation, the cells were pelleted and analyzed in FACSCalibur Flow Cytometer (BD Bioscience, USA). Annexin V^+^/PI^−^ (lower right) cells were recognized as apoptotic cells, while Annexin V^+^/PI^+^ (upper right) cells were defined as necrotic (or late apoptotic). The data were analyzed using FACSComp5.2.1 Analysis Software (Becton-Dickinson Company, USA). Similar tests were performed on K562 cells after 48 h treatment and on KB after 72 h treatment.

### 3.3. Statistics

All data were expressed as mean ± SD Student *t*-test was used to compare the data of control group with treated group. The condition of *P* < .05 was considered to be statistically significant.

## 4. Results

### 4.1. Anticancer Activity of the Isolated Fractions

The aim of an anticancer agent is to trigger the apoptosis signaling system in these cancer cells whilst disturbing their proliferation [11, 12]. Many *in vitro* human cancer cells models, including K562 [1] and KB [13], have been used to screen herbal extracts for anticancer activity.

**Figure 1:**
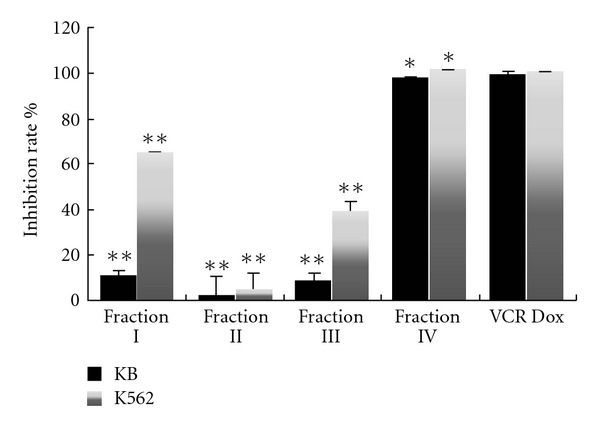
Anticancer effect of fractions I, II, III, and IV on KB and K562 cell lines. The cells were incubated with vincristine sulfate (VCR) or doxorubicin hydrochloride (Dox) or other test compounds for 48 h. Incubation with DMSO alone as control and the final concentration of DMSO in the medium never exceeded 0.1%. After 48 h, the MTT assay was performed to measure cell inhibition rate. Results were reported as mean (*n* = 4) percent of untreated control with error bars showing the standard deviation. Asterisks indicate significant cytotoxicity relative to the positive control (*P < .05, **P < .01).

**Figure 2:**
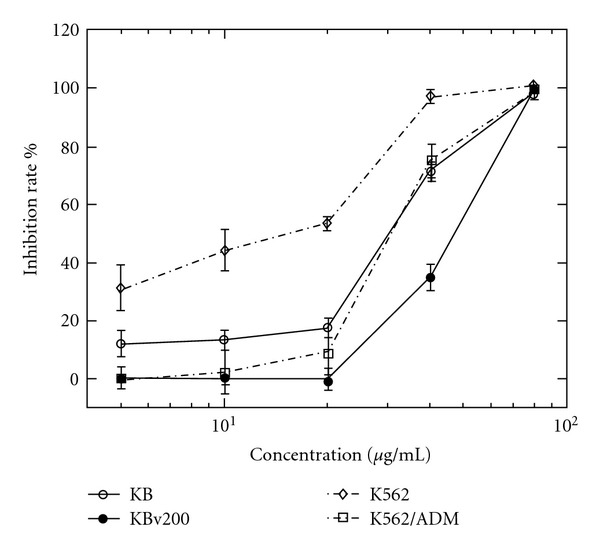
Anticancer activity of fraction IV on different cell lines. Results were expressed as means ± SD of four separate experiments for each data point.

In the present work, the anticancer activity of fraction I, II, III, and IV was assessed using the MTT assay on the human oral carcinoma line KB and human erythroleukemia cell K562. The results were exhibited as the inhibition rate for each fraction tested on KB and K562 cell lines (Figure 1). Fractions I, II, and III just displayed a weak cytotoxicity on KB cells. Fraction I and III showed moderate inhibition effect on K562 cells, while fraction II did not inhibit the growth of K562 cells. However, fraction IV showed significant cytotoxicity on the cancer cells after 48-h exposure, inhibition rate was above 90% of K562 and KB at 80 *μ*g mL^−1^. The positive cytotoxic control agent, vincristine sulfate (2 × 10^−4^ M) and doxorubicin hydrochloride (1.4 × 10^−*­*5^ M), can cause significant cytotoxicity at the very low concentration. The inhibition rate of fraction IV was above 80% compared with control groups and was in a dose-dependent manner in a range of 5–80 *μ*g mL^−1^ (Figure 2). It, therefore, suggests that fraction IV is a promising candidate for further investigation.

### 4.2. Identification of Active Compounds in Fraction IV

**Figure 3:**
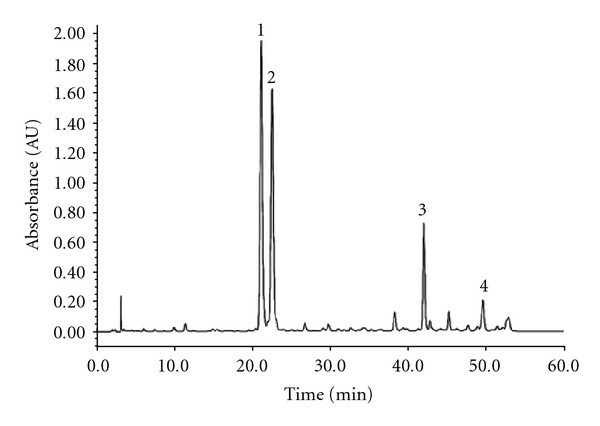
HPLC chromatogram of the *Psoralea corylifolia* L. fraction IV, detection wavelength 300 nm. Peaks are described in the text and putative identifications are given in Table 1.

To screen bioactive compounds from fraction IV, HPLC-API-MS^n^ was employed to identify the components.  Figure 3 showed the analytical result of the chromatographic separation. Molecular weights of four major peaks were obtained. By comparing API-MS^n^ data, coupled with ultraviolet absorption, HPLC retention time with data from [3, 13, 14], four possible structures of these compounds were dereplicated. The positive ionization mode was used, so most of the *m*/*z* data are [M + H]^+^. In this ionization mode, we can deduce whether the substance have isopentenyl from the *m*/*z* data [M-55]^+^, because flavones and coumarin combined with isopentenyl would lose this group and indicate such *m*/*z* data. Because most of flavones and coumarins have a keto carbonyl group, their *m*/*z* data are often characterized by [M–C_4_H_7_]^+^ and [M + H–CO]^+^, there would be some [M-55]^+^, [M + H-28]^+^ and [M-55-28]^+^ fragments [3]. The detailed data of each peak are listed in Table 1.

### 4.3. Dose-Dependent Anticancer Activity of Psoralen and Isopsoralen

**Figure 4:**
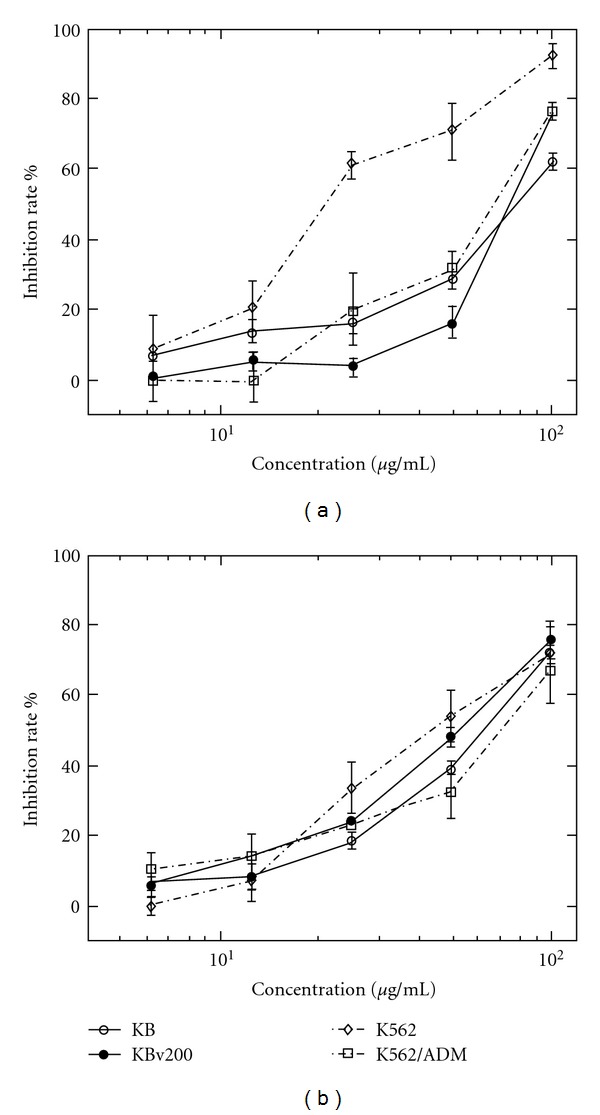
The inhibition ability of psoralen (a) and isopsoralen (b) on proliferation of four tumor cells. The cells were incubated with vincristine sulfate or doxorubicin hydrochloride and different concentrations of psoralen and isopsoralen for 48 h. Incubation with DMSO alone as control and the final concentration of DMSO in the medium never exceeded 0.1%. After 48 h, the MTT assay was performed to measure cell inhibition rate. Results are expressed as means ± SD of four separate experiments for each data point. Significant difference was set at .95.

The major effective components of this herb are coumarins, in which psoralen and isopsoralen are two major compounds. Pharmacological test revealed that psoralen and isopsoralen have anticancer [15], immunomodulatory [7], antibacterial and antivirus properties [2, 15]. Psoralen derivatives are also active *in vitro* against human melanoma cell line [16] (Figure 4).

**Figure 5:**
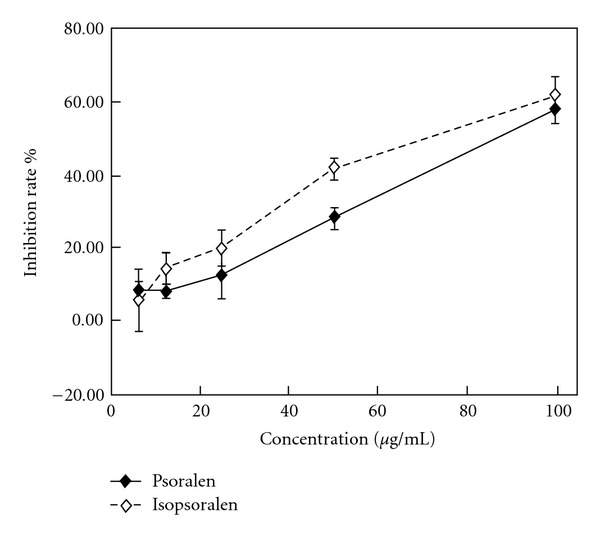
The inhibition ability of psoralen and isopsoralen on proliferation of HPDL cells. The cells were incubated with doxorubicin hydrochloride and different concentrations of psoralen and isopsoralen for 48 h. Incubation with DMSO alone as control and the final concentration of DMSO in the medium never exceeded 0.1%. After 48 h, the MTT assay was performed to measure cell inhibition rate. Results are expressed as means ± SD of four separate experiments for each data point.

In this study, psoralen and isopsoralen were isolated after purification, and the anticancer effects of them on four cell lines were studied. The inhibition rate of psoralen and isopsoralen showed a concentration-dependent manner (Figure 5). IC_50_ (drug concentration resulting in a 50% inhibition of cell growth) value of fraction IV, psoralen and isopsoralen is listed in Table 2.

### 4.4. Effects of Psoralen and Isopsoralen on Cancer Cell Apoptosis

**Figure 6:**
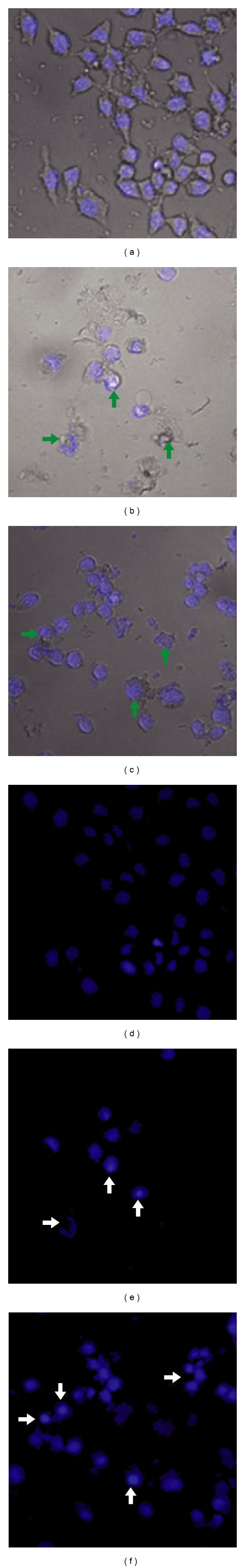
Microscopic analysis of the cytotoxic effects of psoralen and isopsoralen. A group of cells undergoing cell death was examined by fluorescence microscopy after 48 h treatment. After 48 h of exposure, the nucleus of KB cells as well as the cell membrane appears to shrink (green arrows) and the chromatin appears brighter due to condensation (white arrows). Besides membrane blebbing is apparent and the nuclear architecture is rougher in appearance. (a and d): Untreated control KB cells; (b and e): 50 *μ*g mL^−1^ psoralen-treated KB cells; (c and f): 50 *μ*g mL^−1^ isopsoralen-treated KB cells.

The morphological examinations of the KB cells were illustrated using phase contrast microscope and fluorescence microscopy (Figure 6). The control group cells showed a typical polygonal and cobblestone monolayer appearance (Figure 6(a)). Significant decrease of the number of KB cells treated with psoralen (50 *μ*g mL^−1^) or isopsoralen (50 *μ*g mL^−1^) for 48 h was observed compared with the control group. Furthermore, the cell, treated with psoralen and isopsoralen for 48 h began to have morphological changes, showing round-shaped cells poorly adhered to the culture flasks (Figures 6(b) and 6(c)). The changes of nuclear morphology of KB with psoralen or isopsoralen treatment for 48 h were analyzed under a fluorescence microscope by Hoechst-staining (Figures 6(d)–6(f)). The nuclei of the treated cells have nuclear shrinkage and condensed chromatin.

Based on the morphological changes of KB cells after treated by psoralen or isopsoralen for 48 and 72 h, we further verified the occurrence of psoralen and isopsoralen induced apoptosis by Annexin V-PI staining methods. The results showed that the proportion of apoptotic cells (lower right) significant elevated in isopsoralen (50 *μ*g mL^−1^)-treated KB cells from 7.52% in untreated cells to 15.16%, while the psoralen (50 *μ*g mL^−1^)-treatment increased the apoptotic ratio to 9.30%. The psoralen and isopsoralen considerably elevated the proportion of apoptotic cells after 48 h treatment. However, there was no significant effect after 72 h treatment. And there is no difference or even a slight decrease of necrotic cells (upper right) in KB cells after treated with psoralen and isopsoralen for 48 h. These results suggested that apoptosis but not necrosis contributed to the psoralen- and isopsoralen-induced death of KB cells. Similarly, the proportion of apoptotic cells elevated in isopsoralen (50 *μ*g mL^−1^)-treated K562 cells from 6.19% in untreated cells to 14.28%, while the psoralen (50 *μ*g mL^−1^)-treatment increased the apoptotic ratio to 9.87%.

## 5. Discussion and Conclusion

One important source of newer chemotherapeutic agents is those chemicals derived from herbal sources. A number of phytochemicals have been demonstrated to possess anticancer effects [17]. The use of complementary and alternative medicine such as botanical extracts is becoming increasingly popular among cancer patients [18, 19].

In our study, we revealed for the first time the potent cytotoxic actions of *P.corylifolia* L. extract (fraction IV) using two cancer cell lines and their corresponding multidrug-resistant (MDR) cell lines. Further analysis of the constituent reveals that psoralen and isopsoralen are two active constituents in the extract. Fraction IV, psoralen and isopsoralen can inhibit the growth of K562 and K562/ADM at low concentration. They can also inhibit the growth of normal human primary cell HPDL, but the IC_50_ values were higher than ones of tumor cell lines, which suggested these two active components had potential selective cytotoxicity. Specifically, psoralen inhibited the growth of KB, KBv200, K562 and K562/ADM with maximum inhibition rate 62.3, 75.3, 92.4, and 76.6% at 80 *μ*g mL^−1^, respectively; and isopsoralen exhibited a maximum inhibition 72.6, 75.6, 71.6, and 67.0% at the same concentration. It was interesting to find that the IC_50_ value of fraction IV was lower than both psoralen and isopsoralen. One plausible explanation is that psoralen and isopsoralen have a synergic cytotoxic effect toward cancer cells. Or there may be other active constituents, which possess a stronger efficacy against cancer cells in fraction IV. A predominant property is that fraction IV, psoralen and isopsoralen do not show significant cross resistance in the MDR cell line KBv200 and K562/ADM. Resistance factor [20] of fraction IV, psoralen and isopsoralen is 1.13, 0.98, and 0.80 on KBv200, respectively, and 2.69, 2.57 and 1.45 on K562/ADM. This indicates they may be poor substrates for multidrug resistance transporters, for example, P-gp, MRP, in cancer [20, 21] and can circumvent the MDR mechanism in curing cancer.

The pharmacological mechanism of the anticancer activity of *P. corylifolia* L. extract, psoralen and isopsoralen is still not fully understood. Previously psoralen had been reported to destroy the mitochondria, and decrease the energy supply for cancer cells and inhibiting the synthesis of nucleic acid and protein [15]. Nicoletta et al. [22] reported that psoralens have been proposed and used for psoriasis, vitiligo and mycosis fungoides (T-cell lymphoma). Our study showed that psoralen and isopsoralen induced cancer cell apoptosis. Cell apoptosis is mediated by many factors. P53 is a transcription factor at the pivot of a number of pathways that mediate apoptosis in response to a variety of cellular stresses [23]. The differences of apoptotic response to psoralen and isopsoralen may provide some information such as whether or not the induction of apoptosis of *P. corylifolia* L. extract is dependent on p53.

In summary, analytical and pharmacological data obtained from this study suggest that *P. corylifolia* L. significantly inhibits proliferation of sensitive and MDR cancer cells *in vitro*, psoralen and isopsoralen are responsible for the anticancer activity. The mechanism of apoptosis induction and the synergic cytotoxic effect will be the focus of future studies, because the seed of *P. corylifolia* L. is a commonly used botanical medicine.

## Funding

Chinese National Basic Research Priorities Program (No. 2005CB523402); Program for New Century Excellent Talents in University (NCET-06-0515).

## Figures and Tables

**Table 1:** Putative identification of main components of *Psoralea corylifolia* L. fraction IV.

Peak No.^a^	Mass data	UV (nm)	Identification
1	187 [M + H]^+^	212.0, 240.0, 296.0, 326.0	Psoralen^b^
2	187 [M + H]^+^	204.0, 244.0, 300.0	Isopsoralen^b^
3	323 [M + H]^+^, 267 [M–C_4_H_7_]^+^, 239 [M–C_4_H_7_–CO]^+^	208.0, 248.0	Neobavaisoflavone
4	321 [M + H]^+^	248.0, 300.0	Corylin

^a^Peaks No. refers to Figure 3;

^b^Identification throguh comparison the retention time with that of standard substance.

**Table 2:** Cytotoxic activity of fraction IV, psoralen and isopsoralen against cancer cell lines.

	Cell line					
	KB	KBv200		K562	K562/ADM	
	IC_50_ (*μ*g/mL)	IC_50_ (*μ*g/mL)	RF^a^	IC_50_ (*μ*g/mL)	IC_50_ (*μ*g/mL)	RF^a^
*Psoralea corylifolia* L. fraction IV	21.60	24.40	1.13	10.00	26.90	2.69
Psoralen	88.10	86.60	0.98	24.40	62.60	2.57
Isopsoralen	61.90	49.40	0.80	49.60	72.00	1.45

^a^Resistance factor (RF) was calculated as RF = IC_50_(R)/IC_50_(S); S, sensitive cell lines (KB, K562), R, multidrug-resistant cell lines (KBv200, K562/ADM), respectively.
